# Nano Boron Oxide and Zinc Oxide Doped Lignin Containing Cellulose Nanocrystals Improve the Thermal, Mechanical and Flammability Properties of High-Density Poly(ethylene)

**DOI:** 10.3390/polym16010036

**Published:** 2023-12-21

**Authors:** Dilpreet S. Bajwa, Greg Holt, Nicole Stark, Sreekala G. Bajwa, Saptaparni Chanda, Mohiuddin Quadir

**Affiliations:** 1 Mechanical and Industrial Engineering Department, Montana State University, Bozeman, MT 59717, USA; saptaparni.chanda@student.montana.edu; 2 Cotton Production and Processing Research Unit, United States Department of Agriculture, Agricultural Research Service, Lubbock, TX 79403, USA; greg.holt@usda.gov; 3 Forest Biopolymer Science and Engineering, United States Department of Agriculture, Forest Service, Forest Products Laboratory, Madison, WI 53726, USA; nicole.stark@usda.gov; 4 College of Agriculture, Montana State University, Bozeman, MT 59717, USA; sreekala.bajwa@montana.edu; 5 Department of Coatings and Polymeric Materials, North Dakota State University; Fargo, ND 58108, USA; mohiuddin.quadir@ndsu.edu

**Keywords:** lignin-coated cellulose nanocrystal, nano zinc oxide, nano boron oxide, mechanical property, thermal property, fire resistance

## Abstract

The widely used high-density polyethylene (HDPE) polymer has inadequate mechanical and thermal properties for structural applications. To overcome this challenge, nano zinc oxide (ZnO) and nano boron oxide (B_2_O_3_) doped lignin-containing cellulose nanocrystals (L-CNC) were blended in the polymer matrix. The working hypothesis is that lignin will prevent CNC aggregation, and metal oxides will reduce the flammability of polymers by modifying their degradation pathways. This research prepared and incorporated safe, effective, and eco-friendly hybrid systems of nano ZnO/L-CNC and nano B_2_O_3_/L-CNC into the HDPE matrix to improve their physio-mechanical and fire-retardant properties. The composites were characterized using Fourier transform infrared spectroscopy, scanning electron microscopy, energy dispersive X-ray analysis, thermo-gravimetric analysis, differential scanning calorimetry, dynamic mechanical analysis, horizontal burning test, and microcalorimetry test. The results demonstrated a substantial increase in mechanical properties and a reduction in flammability. The scanning electron microscope (SEM) images showed some agglomeration and irregular distribution of the inorganic oxides.

## 1. Introduction

The effect of fossil fuel-derived plastics can be mitigated using biobased polymers, composites, or fillers [1]. Functional polymer composite materials are one of the flagship products for researchers and the industrial sector. The incorporation of fillers or nanofillers in the polymer matrix creates physical cross-link points which can improve the viscoelastic and mechanical properties of the composites [2]. However, the dispersion and distribution of nanofillers in the polymer matrix controls the overall properties of the composites. There are studies that evidenced the modification of the polymer properties by the incorporation of biobased nanofillers [1]. Among biobased fillers, cellulose nanocrystals (CNCs), which can be obtained by the acid hydrolysis of cellulose fibers, are one of the most potential nanofillers due to their excellent mechanical properties, high aspect ratio, low density, and biodegradability [3]. There are several studies [4] that showed the modification of processing and physical properties of a polymer composite by incorporation of CNCs. However, the hydrophilic nature of CNCs promotes the formation of agglomerates in a hydrophobic polymer matrix and intermolecular hydrogen bonds due to the presence of hydroxyl groups on its surface. Those agglomerates act as stress concentration points which lead to the early failure of the composite. Due to these reasons, CNC-incorporated composites are still not commercialized [5]. Several approaches such as solution mixing, reactive compatibilization, ring-opening polymerization, and surface modification of CNC have been taken to improve the dispersion and interfacial adhesion of CNCs within the polymer matrix [2]. But the harsh chemicals used in the surface modification processes affect CNCs’ intrinsic properties and increase the overall cost of the material. The addition of a compatibilizing material such as a surfactant, starch, lignin, etc., can be an alternative solution to improve the dispersion of CNCs in a polymer matrix. Lignin is the most interesting material because it is naturally present in biomass. Lignin is a complex, three-dimensional aromatic polymer that contributes to the various properties of plant cell walls, including mechanical resistance, hydrophobicity, and dimensional stability [6]. Moreover, lignin can absorb UV light, and therefore, transparent coatings with controlled UV-absorbent properties have been developed [7].

There are several studies [8,9,10] that reported improved interaction between CNC and hydrophobic polymers in the presence of lignin. The use of lignin-coated CNCs (L-CNC), to modify the rheological and thermo-mechanical properties of poly (lactic acid) (PLA) composites was reported by Gupta et al., 2017. The lignin coating on CNCs not only improved the dispersion of CNCs but also enhanced their interfacial interaction with the PLA matrix, resulting in a significant improvement in rheological and thermo-mechanical properties. The improvement in mechanical properties can be attributed to a significant increase in the degree of crystallinity of the PLA. Excellent dispersion and compatibility of L-CNCs with PLA allowed the generation of a high density of nucleating sites resulting in an increase in the degree of crystallinity of the PLA matrix. Improvement in the storage modulus at higher loading of L-CNCs can be attributed to both high crystallinity and reinforcement by L-CNCs [8]. In another article by Wei et al., 2017, L-CNCs were compounded with PLA via melt processing. High Young’s modulus and elongation at break values were obtained by the addition of 2% L-CNCs to PLA. Better interfacial bonding of more hydrophobic L-CNCs and hydrophobic PLA matrix was achieved [9]. L-CNC was incorporated by Feng et al., 2017 into methacrylate (MA) resin. Mechanical properties increased with the addition of 0.1% and 0.5% L-CNC, and thermal stability was improved at 0.5% L-CNC. The dynamic mechanical analysis demonstrated that the incorporation of L-CNC increased the storage modulus in the rubbery plateau [10].

With the advent of nanotechnology, nanosized fire retardant particles have modified the degradation pathway of the polymer matrix and also changed their rheological behavior. Inorganic oxides are very popular members of the flame-retardant group because of their low toxicity and low smoke evolution. ZnO-coated cellulose fabrics have shown exceptional flame retardancy [7]. Textiles and upholstery cotton (cellulose) materials are now being treated with nano-sized inorganic particles to impart fire resistance [11,12]. Recently, nano ZnO formulation using polycarboxylic acid was added as a flame retardant for cotton and its blend. In the last ten years, ZnO, micro ZnO, and nano ZnO have gained a lot of attention as fire retardant materials for different synthetic polymeric-based materials, cellulosic cotton, and other lingo-cellulosic fibers [11]. Boron-based compounds are a rapidly developing new class of fire-retardant materials. Under fire exposure, boric acid provides a glass-like coating on the exposed surface inhibiting the flow of oxygen to the exposed polymer surface, promoting polymer dehydration and char formation [13]. Boron is also used to reduce flame spread rate, but it can also stimulate smoldering. However, boric acid is used synergistically with boron to suppress smoldering [14]. The low toxicity, eco-friendliness, preservative effectiveness, and neutral pH are some of the beneficial effects of boron-based compounds [15].

In the current research, L-CNCs were coated with ZnO and B_2_O_3_ as fire-retardant additives and dispersed in the high-density poly (ethylene) (HDPE) matrix using a melt blending extrusion process. The thermo-mechanical and fire-retardant properties of the manufactured composites were studied. Overall, the main aim of the research was to improve the dispersion of CNC in the hydrophobic polymer matrix in the presence of lignin and enhance the thermal, physical, and mechanical properties of the composites.

## 2. Materials and Methods

### 2.1. Materials

High-density poly (ethylene) (HDPE) powder with a melt index of 3.5 g/10 min, and a melting temperature of 126 °C, purchased from Nova Chemicals was used as the polymer matrix. The L-CNC contains 2 wt% of lignin with dimensions of 10–15 nm width and 80–100 nm length and hence, aspect ratios of 5–10 were provided by USDA Forest Products Laboratory Madison, WI, USA. Zinc acetate dehydrate and sodium hydroxide were purchased from Sigma Aldrich for producing Zinc oxide (ZnO). Boron oxide powder and oleic acid were also purchased from Sigma Aldrich to produce nano boron oxide. Methanol was purchased from the chemistry store of MSU and used as a solvent for nano ZnO production. Deionized (DI) water purchased from Millipore, USA was used as the solvent.

### 2.2. Synthesis of ZnO Coated L-CNC

Nano ZnO was prepared using the previously reported method [16]. Zinc acetate dehydrate (0.02 mol) was dissolved in 50 mL of methanol and heated at 50 °C under vigorous stirring for 0.5 h to make a precursor solution A. Then, 0.04 mol of sodium hydroxide was dissolved in 50 mL of methanol and heated at 50 °C along while vigorously stirred for 1 h, to make precursor B. To make ZnO nano-sol, solution B was added into solution A, drop-wise under constant stirring for 0.5 h at 50 °C. The mixture was stirred for another 2 h and cooled at room temperature to obtain transparent ZnO sol. This ZnO sol was freeze-dried and added to aqueous L-CNC dispersions at different weight ratios (1:1, 1:2, 2:1). After 4 h of mixing, the blends were freeze-dried for use in polymer composites.

### 2.3. Synthesis of B_2_O_3_ Coated L-CNC

Nano boron oxide was prepared using the previously reported method [17]. Boron oxide (B_2_O_3_) granules (0.100 g or 1.43 mmol) were grounded to a coarse powder using an agate mortar and pestle. This powder was transferred to a 50 mL centrifuge tube and 5 mL (35 mmol) of oleic acid was added. The reaction mixture was then probe sonicated at 50 unit amplitude for 3 h while placed in an ice bath. Following this, the colloidal product obtained was centrifuged at 9000 rpm for 15 min and the supernatant oleic acid was discarded. The solid product was washed with 5 mL of anhydrous hexane and vacuum-dried in an oven to produce a fluffy, white powder. This B_2_O_3_ powder was added to aqueous L-CNC dispersions at different weight ratios (1:1, 1:2, 2:1). After 4 h of mixing, the blends were freeze-dried for use in polymer composites.

### 2.4. Design and Manufacture of Polymer Composites Using Inorganic Oxide Coated L-CNC as Filler Material

HDPE served as the matrix resin to manufacture composite samples (Table 1). Three ZnO-L-CNC formulations synthesized in the previous step were used to manufacture HDPE masterbatches. Freeze-dried ZnO-L-CNC was melt-blended with resin. Each master batch was further used to manufacture test planks representing 1:1, 1:2, and 2:1 ZnO-L-CNC ratios. The design of the experiment included three types of ZnO-L-CNC complexes, pristine L-CNC incorporated, and control (only HDPE) with five replications. The B_2_O_3_ incorporated samples were prepared by using the same weight ratios as ZnO incorporated samples. The smaller size and higher surface area of the inorganic oxides facilitated their lower add-on percentage to the composites [11,18]. Due to their smaller size, the inorganic oxides can impart a similar level of fire retardancy at a much lower add-on percentage compared to their bulk counterparts. Another important reason behind using a lower add-on percentage of the inorganic oxides is their scissoring effect in the presence of high temperature and high shear atmosphere. The inorganic oxides can degrade the polymer matrix during the extrusion process, which is not desirable [11,12]. Composite samples were prepared by melt compounding in a Thermo Fisher Scientific HAAKE Minilab II dual screw extruder. Samples were extruded at 100 rpm and 145 °C for 5 min. After mixing, composites were injection molded using a Thermo Fisher Scientific Minijet Pro injection molder. Samples were molded into either a DMA sample mold (Thermo Fisher Scientific, Waltham, MA, USA, Part # 557-2295) with dimensions of 60 mm × 10 mm × 1 mm or a tensile test dog bone mold (Thermo Fisher Scientific, Waltham, MA, USA) of the geometry described in ASTM D1708 [19].

### 2.5. Characterization

#### 2.5.1. Fourier Transform Infrared Spectroscopy (FTIR)

FTIR spectroscopy Nicolet i550, (Thermo Fisher Scientific, MA, USA) was used to analyze the chemical structure of the composites in an attenuated total reflection (ATR) mode. The final spectrum of each sample was an average of 32 scans at a resolution of 4 cm^−1^, in the wavenumber range of 400–4000 cm^−1^.

#### 2.5.2. Scanning Electron Microscopy

The composites were cryo-fractured in the liquid nitrogen atmosphere and the morphology of the cross-section of the composites was analyzed using field emission scanning electron microscopy (FESEM). Images of cross-section morphology were taken at different magnifications using a field emission scanning electron microscope Supra 55VP (Zeiss, Thornwood, NY, USA), operating at an accelerating voltage of 15.0 kV, in the Image and Chemical Analysis Laboratory (ICAL) at Montana State University. The samples were coated with a thin film of Iridium for 60 s for conductivity before capturing the images.

#### 2.5.3. Thermogravimetric Analysis

Thermogravimetric analysis (TGA) was carried out to analyze the thermal stability of the composites using a thermogravimetric analyzer TGA Q5000 (TA Instruments, New Castle, DE, USA) at Montana State University. The samples were tested in thermal degradation mode, under a flowing nitrogen atmosphere with a flow rate of 60 mL/min, from 30 °C to 600 °C, at a heating rate of 10 °C/min.

#### 2.5.4. Differential Scanning Calorimetry (DSC)

The crystallization ability of the composites was studied using DSC. The melt peak, crystallization peak, and fusion enthalpy of the samples were measured by using a DSC Q2500 (TA Instruments, New Castle, DE, USA) under a nitrogen atmosphere at Montana State University. The samples of about 4–5 mg were taken and scanned over a temperature range from 30 °C to 400 °C at a heating rate of 10 °C/ min.

#### 2.5.5. Dynamic Mechanical Analysis

The viscoelastic properties of the composites were studied using a Dynamic mechanical analyzer Q800 (TA Instruments, Q2500, New Castle, DE, USA) to measure the dynamic storage and loss modulus of the samples, using a three-point bending fixture. The composites were heated from 30 °C to 110 °C, with a frequency of 1 Hz, amplitude of 20 μm, and a force track of 125%. Two replicates were tested for each formulation and the mean values were reported.

#### 2.5.6. Tensile Testing

The tensile properties of the composites were measured using an MTS C43 load frame with 5 mm/min speed and a 35 mm gauge length from clamp to clamp (MTS Systems, Eden Prairie, MN, USA). Five replicates were tested for each formulation and the mean values were reported.

#### 2.5.7. Horizontal Burning Test

The horizontal burning test was carried out to obtain a preliminary idea of the fire-retardant behavior of the composites. The test was conducted on a rectangular bar specimen with dimensions of 60 mm × 10 mm × 1 mm according to the ASTM D635-14 standard [20]. The samples were fixed to the mount and their longitudinal axis was kept in the horizontal way during the test. A butane lighter was used, and the flame was applied to the free end of the sample at a 45° angle. For each formulation, four replicates were tested, and the mean values were reported.

#### 2.5.8. Microcalorimetry Test

Microcalorimetry test of the composites was carried out to evaluate the heat release properties which is essential for the fire-retardant behavior of composites. The static and dynamic combustion parameters of the composites were measured using a Microscale Combustion Calorimeter (MCC-2) by Deatak (University of Texas, Austin) following ASTM D7309 standard [21]. Sample weighing 2–3 g was combusted by rapid-controlled pyrolysis under an atmosphere of 20–80% oxygen to nitrogen gas ratio. The combustor temperature was set to 900 °C with a heating rate of 1 °C/min. For each formulation, three replicates were tested, and the mean values were reported.

## 3. Results and Discussion

### 3.1. Spectroscopy Analysis of the Composites

**Figure 1:**
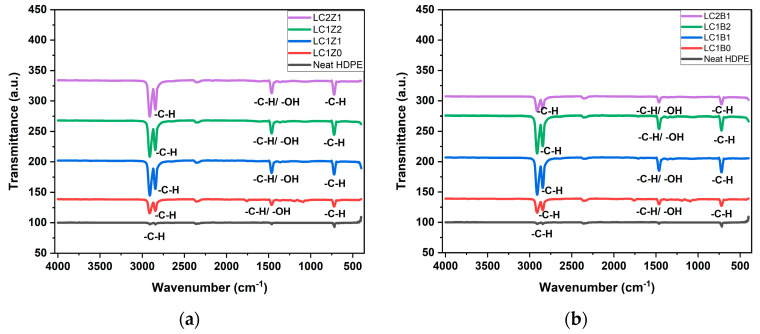
Transmission spectra of (**a**) ZnO-coated L-CNC composites and (**b**) B_2_O_3_ coated L-CNC composites.

The FTIR spectra of neat HDPE and all the composites containing ZnO and/or B_2_O_3_ are shown in Figure 1. The neat HDPE spectra showed characteristic asymmetric and symmetric stretching bands of -C-H, respectively, at 2917 cm^−1^ and 2852 cm^−1^. The bending vibration modes of CH_3_ and CH_2_ rocking vibration modes were also observed at 1472 cm^−1^ and 720 cm^−1^, respectively [16]. In the case of L-CNC incorporated composites, the -C-H vibration peaks of -CH_2_ and -CH_3_ groups at 2914 cm^−1^ and 1461 cm^−1^ of lignin overlapped with the -C-H bending vibration peaks of PE and increased the intensity of the peaks. The -C-H vibration of -CH_3_O groups of lignin showed an absorption band at 2844 cm^−1^ which also overlapped with the stretching band of -C-H from HDPE [18], showing the incorporation of lignin in the CNC structure. All the ZnO and B_2_O_3_ incorporated samples showed slightly higher intensity peaks which can be related to the strong interaction between the oxygen groups and inorganic oxide nanoparticles and hydroxyl groups of L-CNC [8].

### 3.2. Morphological Analysis of the Composites

**Figure 2:**
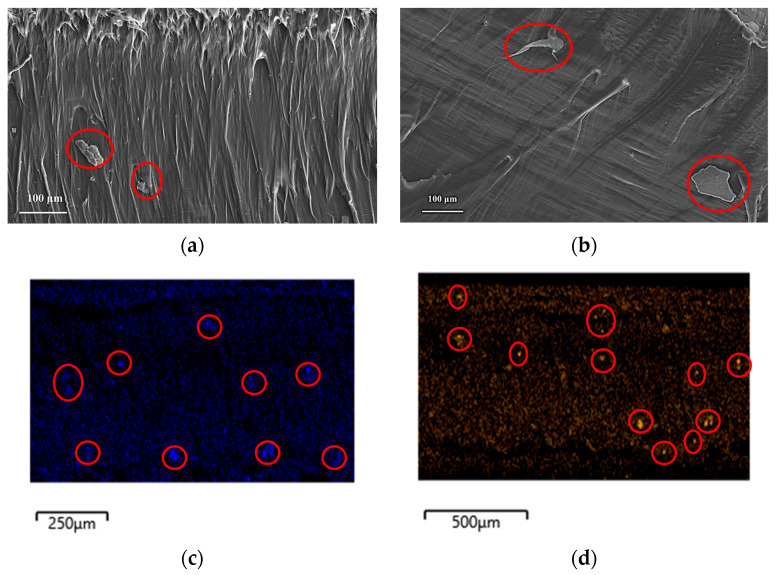
SEM images of HDPE composites cross-sections: (**a**) L-CNC-ZnO, (**b**) L-CNC- B_2_O_3_ (agglomerations shown in red circles) (**c**) elemental mapping of Zn, and (**d**) elemental mapping of B (shown in red circles).

Figure 2 shows the SEM micrographs of the cryo-fractured cross-sections of the ZnO and B_2_O_3_ treated composites. The micrographs showed some aggregation which may be the result of CNC/inorganic nano oxide complexes. The presence of ZnO and B_2_O_3_ was confirmed by energy dispersive X-ray (EDX) analysis and the images showed an irregular distribution of inorganic oxide nanoparticles.

### 3.3. Thermogravimetric Property Analysis of the Composites

The effect of ZnO and B_2_O_3_ incorporation on the thermal properties of the HDPE composites was investigated using thermogravimetric measurement and represented in Table 2. All formulations demonstrated a single-step degradation curve. T_onset_, T_50_, T_endset_ values are used to analyze the thermal stability of the composites. The initial degradation temperature is represented by T_onset_ at which the composites start degrading, T_50_ is the temperature at 50% weight loss and the temperature at the end of degradation is called T_endset_.

The thermal degradation of lignin occurred over a broad range of temperatures, from 150 °C to 800 °C, resulting in lignin structure decomposition and leading to the formation of a highly stable carbonaceous structure. The thermal stability of lignin is strongly dependent on its chemical structure and specific chemical composition [8]. The presence of lignin generally leads to a decrease in material thermal stability due to catalysis of thermal degradation at lower temperatures. The scissoring effect of ZnO also played a significant role in degrading the thermal properties of the composites [11].

A similar trend was observed in the case of B_2_O_3_-coated L-CNC composites. The decreasing trend of thermal stability of B_2_O_3_ coated L-CNC composites can be attributed to the inherent lower thermal stability of lignin along with oleic acid present in B_2_O_3_ structure. To prevent the highly hygroscopic nature and moisture adsorption capability of B_2_O_3_, oleic acid was introduced on its surface. The moisture contamination can create microvoids in the system which in turn can reduce the thermo-mechanical properties of the composites [12]. But the lower flash point of oleic acid (189.9 °C) [22] and the inherent lower thermal stability of lignin adversely affected the overall thermal stability of the composites. The residue weight for ZnO and B_2_O_3_-containing composites was significantly higher than neat HDPE. It demonstrated that metal oxide-containing samples can improve the thermal stability of thermoplastic polymers.

### 3.4. Crystallization Behavior Analysis of the Composites

**Figure 3:**
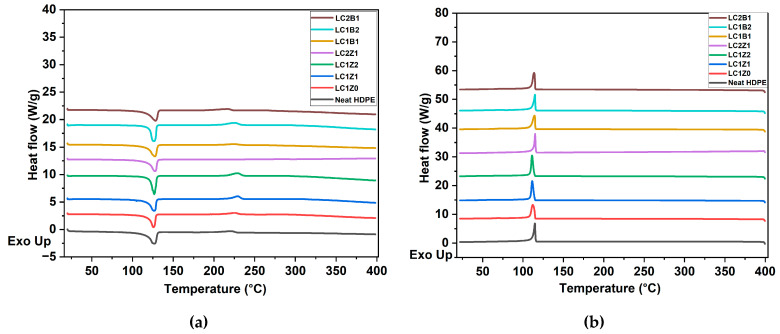
DSC thermograms of (**a**) heating cycle and (**b**) cooling cycle of the composites.

DSC was used to study the non-isothermal crystallization behavior of the HDPE composites. The DSC results are shown in Figure 3, and Table 3. The crystallinity (Xc) was determined by the following equation:

X_c_ (%) = ΔH_m_ × 100/ ΔH_m_^0^ × (1 − x) (1)

where, ΔH_m_ is the melting enthalpy, ΔH_m_^0^ is the theoretical enthalpy of 100% crystalline HDPE (293 J g^−1^), and x is the weight fraction of filler (L-CNC-ZnO or B_2_O_3_).

Incorporation of L-CNC into the system decreased the crystallinity (%) (Xc = 36.45%) compared to neat HDPE. The reason behind this observation may be the formation of less stable random crystals in the presence of L-CNCs. Due to the incorporation of L-CNCs as the nanofiller in the system, heterogeneous nucleation started which in turn reduced the free energy barrier. The confinement effect of the nanocrystals hindered the macromolecular chains diffusing in the lattice and thereby facilitated the formation of disordered and less stable crystal structures [22]. The addition of ZnO to the system did not show any change in crystallization behavior. The decrease in Xc of all the composites compared to neat HDPE can be attributed to the decrease in aspect ratios of fillers during freeze drying, which led to agglomeration, as reported in the literature [23]. The SEM micrographs also showed evidence of large agglomerates in the system. These agglomerates hindered the formation of stable crystals and thus the crystallinity (%) decreased [24]. The small peak observed after the melting point was possibly due to the occurrence of relaxation or molecular arrangement of the polymer matrix [22].

A similar trend was observed in the case of B_2_O_3_-coated L-CNC composites. In the case of B_2_O_3_-coated L-CNC composites, the large and bulky structure of oleic acid present in the B_2_O_3_, along with L-CNC formed agglomerates in the matrix, which hindered the formation of stable crystals [23], thereby decreasing the crystallinity.

### 3.5. Dynamic Mechanical Property Analysis of the Composites

**Figure 4:**
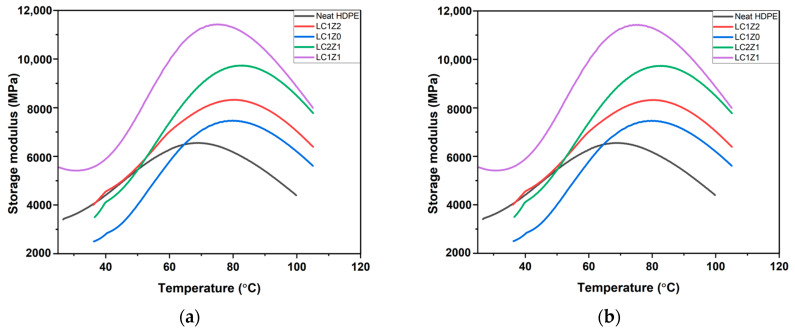
Representative curves of storage modulus as a function of temperature of (**a**) ZnO-coated L-CNC composites and (**b**) B_2_O_3_-coated L-CNC composites.

Dynamic mechanical analysis was used to study the dynamic viscoelastic properties, storage modulus (E′), loss modulus (E″), and loss factor (damping parameter, tan δ). The results are shown in Figure 4. The dynamic mechanical properties of the composites are largely dependent on the filler content. An α-transition peak was observed in the storage modulus curve of neat HDPE around 69.25 °C. Typically for a semi-crystalline polymer, this α-transition is related to the long-range segmental motion, known as transition temperature [13]. All the composites showed a higher transition temperature, around 78 °C, compared to neat HDPE. Insertion of the bulky side group of lignin increased the transition temperature of the composite system due to the restricted mobility of the polymer chains in the amorphous region due to the confinement effect [14]. As the temperature exceeded the transition region, a sharp drop in modulus was observed. The mobility of the HDPE chains in the amorphous region increased leading to decreased modulus [15].

The incorporation of L-CNC (no inorganic oxide) into the system (LC1Z0) as a filler did not improve the storage modulus of the composites. The scenario is the same for LC1Z1 composites.

The inorganic oxide incorporated composites and neat HDPE did show some statistically significant differences in storage modulus. At higher temperatures all the composites with L-CNC and inorganic oxide showed higher storage modulus than neat HDPE. The presence of lignin impacted the dispersion of L-CNCs in the system due to the agglomeration during freeze drying [10], which perhaps impacted the mechanical properties of the composites. In general, the inorganic oxide incorporated composites did not show any statistically significant difference in storage modulus between themselves.

### 3.6. Tensile Property Analysis of the Composites

The mechanical property analysis such as elastic nominal stiffness (k), and elongation at break were investigated to gain an insight into the tensile behavior of the composites, the effect of ZnO and B_2_O_3_ on the tensile properties and comparison of the tensile properties with neat HDPE. Theoretically, the addition of L-CNC to a polymer matrix as a filler should increase and improve the tensile properties of the composites [9]. But, like all the nanofillers, there was some difficulty with uniformly blending L-CNC with the polymer [9]. Eventually, the increase in the amount of L-CNC in the system can adversely affect the mechanical properties of the system.

**Figure 5:**
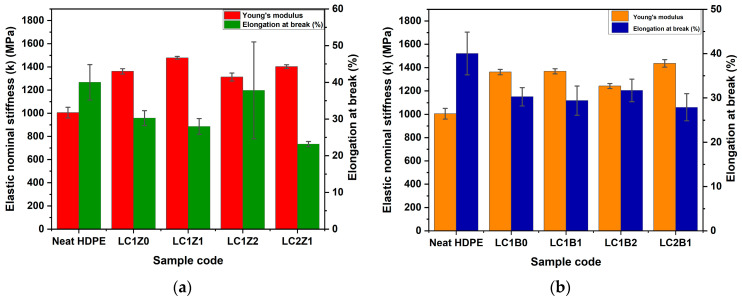
Graphical representation of the tensile properties of (**a**) ZnO-coated L-CNC composites and (**b**) B_2_O_3_-coated L-CNC composites.

In the current research, L-CNC and the inorganic oxides were incorporated in different ratios to observe and analyze their effect on the mechanical properties of HDPE polymer and the results are shown in Figure 5.

Most of the L-CNC incorporated formulations showed a significant improvement in elastic nominal stiffness of the composites, compared to neat HDPE, though no significant change was observed in the case of elongation at break (%). The filler/polymer interaction is more important in the case of low aspect ratio fillers and polymer compared to the higher aspect ratio fillers [9,10]. The incorporation of L-CNC (LC1Z0) in the composites significantly increased the elastic stiffness in comparison with neat HDPE, indicating better interfacial bonding with the HDPE matrix [9]. The incorporation of ZnO in the system further increased the elastic stiffness and LC1Z1 formulation showed the highest value of elastic stiffness compared to the other systems. But no additional improvement in the elastic stiffness was observed in the case of the higher amount of ZnO or L-CNC incorporation. The plausible explanation of this phenomenon can be the agglomeration of the reinforcements (L-CNC/ZnO complex) during freeze-drying, with increasing ZnO concentration.

In addition, the decrease in aspect ratios of the fillers during freeze-drying can change their shape to flakes instead of rod-like shapes due to the agglomeration [25], as shown in the SEM micrographs of the cross-sections of the composites. The agglomerates in the composites can be responsible for the more ductile behavior of the composites and no significant change in the elongation at break (%). Overall, there is around a 30% increase in the elastic stiffness of the ZnO-coated composites compared to neat HDPE. The incorporation of more B_2_O_3_ in the system showed a significant decrease in elastic nominal stiffness compared to ZnO-coated composites. This phenomenon can be related to the micro void formation in the interface of the filler and the matrix [26]. During the production of B_2_O_3_, oleic acid was used as a reaction medium and was capped on the surface of B_2_O_3_ to increase the hydrophobicity. But due to the large and bulky structure of the oleic acid, it had an adverse effect on the tensile properties of the composites. It hindered the interaction between L-CNC and B_2_O_3_, because of the bulky structure of both lignin and oleic acid. Due to this reason, some agglomerates were formed during freeze drying, as shown in SEM micrographs. This lack of interaction between L-CNC and B_2_O_3_ created microvoids at the interfacial region, affecting the tensile properties of the composites [27,28]. Overall, there was around a 27% increase in the elastic nominal stiffness of the composites compared to neat HDPE.

### 3.7. Horizontal Burning Test Analysis

The horizontal burning test was conducted to evaluate the flame spread rate, type of burning, and weight loss (%), to gain a primary idea of the fire-retardant behavior of the composites. The flame spread rate was calculated by recording the time taken by the advancing flame front to cover a pre-defined distance. The thermal degradation of lignin occurred over a broad range of temperatures, from 150 °C to 800 °C, resulting in lignin structure decomposition and leading to the formation of a highly stable carbonaceous structure. The flame spread rate for composites containing lignin-coated CNC with nano zinc oxide varied from 1.04 to 1.19 mm/s and nano boron oxide 0.99–1.07 mm/s was lower than the control neat HDPE which was 1.10 mm/s. The effectiveness of lignin as a fire retardant is strongly dependent on its chemical structure and specific chemical composition [29]. The addition of L-CNCs to HDPE did not show a substantial change in the flame spread rate in the horizontal burning test alone, however, there was a decrease in weight loss (%). The presence of lignin generally leads to a decrease in material thermal stability due to catalysis of thermal degradation at lower temperatures [8]. In this case, the inorganic oxide present in the composites prevented further degradation of the composite structure.

The tests confirmed the formation of char during combustion. The char acts as an insulating layer at the surface of the burning material. The inhibition of the mass transfer of the combustible gases results in the dilution of fuel and subsequent termination of the combustion cycle [30,31]. Because of this reason, there was a decrease in weight loss (%) in both cases of inorganic oxide incorporation as shown in Table 4. Overall, there is an agreement between the mean weight loss (%) during the horizontal burn test and TGA residues, in both tests neat HDPE exhibited the highest weight-loss and lowest char residue confirming the potential of metal oxides as fire retardants.

### 3.8. Microcalorimetry Analysis

The microcalorimetry test was conducted to evaluate the heat release capacity (J/gK), peak heat release rate (W/g), and total heat release (kJ/g) of the L-CNC composite samples and compared to neat HDPE. The heat release rate (HRR) is regarded as the driving force for fire spread and one of the most important properties to control fire. The HRR is calculated by multiplying the weight loss of a material with the heat of combustion of that material [32]. Based on the results of the horizontal burning test and TGA, the five promising formulations were selected for microcalorimetry. Table 5. shows the values for HR capacity, peak HRR, and total HR and their statistical comparison for the neat HDPE, LC1B2, and LC1Z1 formulations.

During the combustion of the composites, the decomposition of the resin matrix is responsible for the release of flammable volatiles. On the other hand, the fillers/fire retardants control the amount of heat released from the polymer composite [33,34].

**Figure 6:**
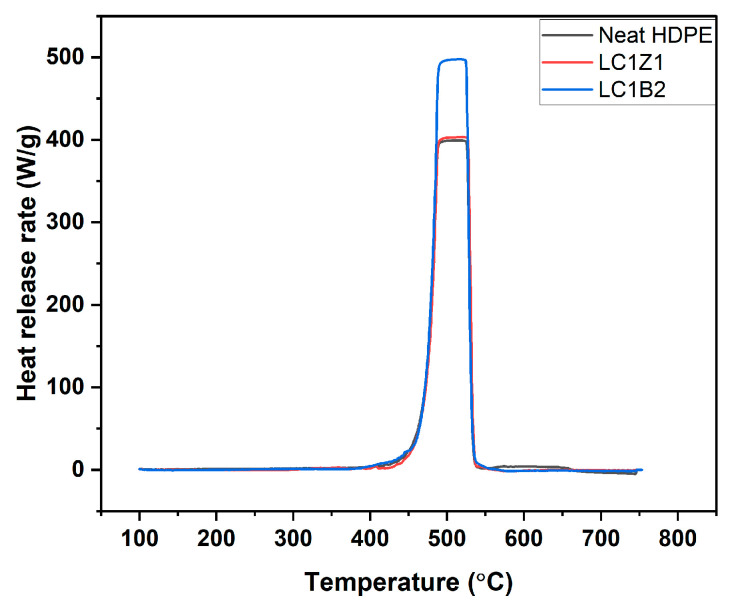
Heat release rate graph of neat HDPE and the composites.

The initial delay period (till 400 °C) before heat release was present because the temperature of the composite was still below the pyrolysis temperature of the matrix, HDPE (Figure 6). In TGA, it was observed that the decomposition of the system started around 400 °C. There was a rapid increase in HRR following the previous induction period where the combustion of the volatiles began at the interface of the composite/fire. Around 500 °C, the PHRR was achieved and after a short stabilization period, the heat release decreased drastically. The shielding effect of the char produced on the exposed polymer surface reduced the penetration of the combustible volatiles, and the low-molecular-weight hydrocarbons, and the decomposition rate of the underlying material declined. The decreasing resin content of the composites also decreased the PHRR [35].

In the case of ZnO coated composites, no statistically significant decrease in HRR and PHRR was observed compared to neat HDPE. The presence of lignin decreased the composites’ thermal stability due to the catalysis of thermal degradation at lower temperatures [8,36]. For B_2_O_3_ coated composites, an increase in PHRR and HRR or a declination of fire-retardant properties compared to HDPE was observed. During the production of B_2_O_3_, the surface of the nanoparticles was covered with oleic acid to increase the hydrophobicity of the inorganic oxides. But the lower flash point of oleic acid (189.9 °C) and the inherent lower thermal stability of lignin adversely affected the overall thermal stability and fire retardancy of the composite system [37,38]. In addition, the large and bulky structures of lignin and oleic acid hindered the interaction between L-CNC and B_2_O_3_. As discussed earlier the aggregates of L-CNC/B_2_O_3_ complex during freeze drying affected the dispersion of L-CNC and B_2_O_3_ in the polymer matrix [39,40] impacting the fire-retardant properties.

## 4. Conclusions

HDPE is one of the most widely used commercial polymers. To improve HDPE’s inadequate thermo-mechanical properties, different types of bio-based nanofillers and fire-retardant materials are incorporated. The current study investigated the role of nano sized ZnO or B_2_O_3_-coated L-CNC as functional additives for improving the fire-resistant behavior and mechanical properties of the HDPE-based composites. The HDPE composite formulations containing nano ZnO or B_2_O_3_ and L-CNC were manufactured using melt blending extrusion and injection molding process. In this study, the presence of inorganic oxides, especially the ZnO-incorporated composites, prevented the adverse effect of the inherent lower thermal stability of lignin. The small size and high surface area of nano ZnO favored the thermal stability of the composites. The TGA, horizontal burning test, and microcalorimetry test supported the aforesaid finding. The B_2_O_3_-coated composites showed a decline in fire properties due to the bulky structure of oleic acid-capped B_2_O_3_, the lower flash point of oleic acid, and the lower thermal stability of L-CNC. The mechanical properties of the composites were improved because of the reinforcement effect of L-CNC and inorganic oxides. There is a 30% increase in elastic nominal stiffness observed in the case of ZnO-coated composites, while the B_2_O_3_-coated composites showed a 27% increase in elastic nominal stiffness compared to neat HDPE. The formation of agglomerates of L-CNC/inorganic oxide complex during freeze drying prevented further improvement in the mechanical properties of the composites. The SEM micrographs showed some agglomerations and irregular distribution of inorganic oxides in the cryo-fractured cross-sections of the B_2_O_3_-containing composites. Therefore, it can be concluded that the mechanical properties of the L-CNC-containing composites were improved due to the reinforcement effect of the L-CNC/inorganic oxide complex compared to neat HDPE. The presence of ZnO in the system prevented degradation of the composite structure compared to B_2_O_3_ incorporated composites due to the presence of oleic acid in the B_2_O_3_ structure. The improvement in the dispersion of the filler in the polymer matrix can further improve the overall mechanical and thermal properties of the system.

## Figures and Tables

**Table 1:** Formulations of composite samples.

Sample Code	Resin (HDPE) (wt%)	LCNC (Weight Ratio)	ZnO/B_2_O_3_ (Weight Ratio)
Neat HDPE	100	-	-
LC1Z0	99	1	0
LC1Z1	99	1	1
LC1Z2	99	1	2
LC2Z1	99	2	1
LC1B0	99	1	0
LC1B1	99	1	1
LC1B2	99	1	2
LC2B1	99	2	1

**Table 2:** Thermal properties of each L-CNC-ZnO and L-CNC-B_2_O_3_ composites.

Sample Name	T_onset_ (°C)	T_50_ (°C)	T_endset_ (°C)	Residue wt. (%)
Neat HDPE	404.05	460.24	481.70	0.5597
LC1Z0	405.91	451.09	469.85	1.487
LC1Z1	380.85	448.53	466.26	3.686
LC1Z2	385.47	450.63	469.12	3.309
LC2Z1	398.21	449.23	466.52	3.463
LC1B0	405.91	451.09	469.85	1.487
LC1B1	387.86	451.36	471.56	3.166
LC1B2	393.61	450.74	470.41	3.414
LC2B1	397.03	455.35	472.91	3.621

**Table 3:** Crystallization behavior of each formulation (L-CNC/ZnO) from DSC.

Sample Name	T_c_ (°C)	T_m_ (°C)	Crystallinity (X_c_) (%)
Neat HDPE	114.54	127.53	44.12
LC1Z0	112.40	125.64	36.45
LC1Z1	111.73	126.61	36.49
LC1Z2	111.56	126.54	37.87
LC2Z1	115.15	127.73	36.75
LC1B0	112.40	126.64	36.45
LC1B1	113.99	127.31	38.47
LC1B2	114.45	126.54	36.69
LC2B1	113.65	128.09	37.65

**Table 4:** Mean flame spread rate and weight loss of ZnO and B_2_O_3_-coated L-CNC composites.

Sample Code	Mean Flame Spread Rate (mm/s)	Mean Weight-Loss (%)
HDPE	1.08 ± 0.26 ^a^	65.77 ± 1.17 ^a^
LC1Z1	1.19 ± 0.14 ^b^	61.35 ± 1.88 ^a^
LC2Z1	1.17 ± 0.055 ^b^	59.68 ± 4.23 ^b^
LC1Z2	1.05 ± 0.035 ^a^	52.37 ± 2.80 ^b^
LC1Z0	0.92 ± 0.005 ^a^	49.78 ± 6.36 ^b^
LC2B1	1.08 ± 0.025 ^a^	61.35 ± 1.88 ^a^
LC1B1	1.02 ± 0.06 ^a^	60.66 ± 0.10 ^b^
LC1B2	0.99 ± 0.04 ^a^	56.04 ± 1.11 ^b^
LC1B0	0.92 ± 0.005 ^a^	53.73 ± 0.62 ^b^

(For the same column, values (means ± SD) followed by the same letter are not significantly different based on Fisher’s least significant difference test at α = 0.05).

**Table 5:** Heat release capacity, peak heat release and total heat release of composites with statistical mean comparison.

Sample Code	HR Capacity (J/gK)	PHRR (W/g)	Total HR (kJ/g)
Neat HDPE	414.67 ^b^	413.03 ^b^	22.73 ^b^
LC1Z1	417.00 ^b^	404.03 ^b^	20.80 ^b^
LC1B2	477.66 ^a^	475.37 ^a^	25.43 ^a^

For the same column, values (means ± SD) followed by the same letter are not significantly different based on Fisher’s least significant difference test at α = 0.05.

## Data Availability

Data available on request.
